# SCORE2 Assessment in the Calculation of Cardiovascular Risk in Patients with Rheumatoid Arthritis

**DOI:** 10.3390/diagnostics11122363

**Published:** 2021-12-15

**Authors:** Iván Ferraz-Amaro, Alfonso Corrales, Belén Atienza-Mateo, Nuria Vegas-Revenga, Diana Prieto-Peña, Julio Sánchez-Martín, Cristina Almeida, Juan Carlos Quevedo-Abeledo, Ricardo Blanco, Miguel Á. González-Gay

**Affiliations:** 1 Division of Rheumatology, Hospital Universitario de Canarias, 38320 Tenerife, Spain; 2 Internal Medicine Department, University of La Laguna, 38071 Tenerife, Spain; 3 Division of Rheumatology, Hospital Universitario Marqués de Valdecilla, Universidad de Cantabria, 39008 Santander, Spain; afcorralesm@hotmail.com (A.C.); mateoatienzabelen@gmail.com (B.A.-M.); nuriavegas2@gmail.com (N.V.-R.); diana.prieto.pena@gmail.com (D.P.-P.); jsm132@hotmail.com (J.S.-M.); rblancovela@gmail.com (R.B.); 4 Epidemiology, Genetics and Atherosclerosis Research Group on Systemic Inflammatory Diseases, Hospital Universitario Marqués de Valdecilla, IDIVAL, 39011 Santander, Spain; 5 Division of Rheumatology, Hospital Doctor Negrín, 35010 Las Palmas de Gran Canaria, Spain; almeidasantiago.cristina@gmail.com (C.A.); quevedojcarlos@yahoo.es (J.C.Q.-A.); 6 Cardiovascular Pathophysiology and Genomics Research Unit, School of Physiology, Faculty of Health Sciences, University of the Witwatersrand, Johannesburg 2000, South Africa

**Keywords:** rheumatoid arthritis, cardiovascular risk assessment, SCORE, SCORE2, carotid ultrasound

## Abstract

Patients with rheumatoid arthritis (RA) are at increased risk for cardiovascular disease (CVD). Risk chart algorithms, such as the Systematic Coronary Risk Assessment (SCORE), often underestimate the risk of CVD in patients with RA. In this sense, the use of noninvasive tools, such as the carotid ultrasound, has made it possible to identify RA patients at high risk of CVD who had subclinical atherosclerosis disease and who had been included in the low or moderate CVD risk categories when the SCORE risk tables were applied. The 2003 SCORE calculator was recently updated to a new prediction model: SCORE2. This new algorithm improves the identification of individuals from the general population at high risk of developing CVD in Europe. Our objective was to compare the predictive capacity between the original SCORE and the new SCORE2 to identify RA patients with subclinical atherosclerosis and, consequently, high risk of CVD. 1168 non-diabetic patients with RA and age > 40 years were recruited. Subclinical atherosclerosis was searched for by carotid ultrasound. The presence of carotid plaque and the carotid intima media wall thickness (cIMT) were evaluated. SCORE and SCORE2 were also calculated. The relationships of SCORE and SCORE2 to each other and to the presence of subclinical carotid atherosclerosis were studied. The correlation between SCORE and SCORE2 was found to be high in patients with RA (Spearman’s Rho = 0.961, *p* < 0.001). Both SCORE (Spearman’s Rho = 0.524) and SCORE2 (Spearman’s Rho = 0.521) were similarly correlated with cIMT (*p* = 0.92). Likewise, both calculators showed significant and comparable discriminations for the presence of carotid plaque: SCORE AUC 0.781 (95%CI 0.755–0.807) and SCORE2 AUC 0.774 (95%CI 0.748–0.801). Using SCORE, 80% and 20% of the patients were in the low or moderate and high or very high CVD risk categories, respectively. However, when the same categories were evaluated using SCORE2, the percentages were different (58% and 42%, respectively). Consequently, the number of RA patients included in the high or very high CVD risk categories was significantly higher with SCORE2 compared to the original SCORE. (*p* < 0.001). In conclusion, although predictive capacity for the presence of carotid plaque is equivalent between SCORE and SCORE2, SCORE2 identifies a significantly higher proportion of patients with RA who are at high or very high risk of CVD.

## 1. Introduction

Patients with rheumatoid arthritis (RA) are at increased risk of cardiovascular (CV) disease [1]. In this sense—an accelerated atherosclerosis process mediated by chronic inflammation—traditional CV disease risk factors together with a genetic component leads to subclinical atherosclerosis [2] and a higher rate of CV events [3] in these patients. Regarding this, it was determined that the presence of subclinical atherosclerosis identified by the presence of carotid plaque using carotid ultrasound predicted the development of CV events and death in patients with RA [4].

Risk chart algorithms often underestimate the risk of CV disease in patients with RA. Such was the case for the Systematic Coronary Risk Assessment (SCORE) [5], which was originally developed for use in the general European population in 2003 and relied heavily on traditional CV disease risk factors. In this sense, the use of noninvasive surrogate markers such as the carotid ultrasound has made it possible to identify RA patients at high risk of CVD who had subclinical atherosclerosis disease and who were included in the low or moderate CVD risk categories when the SCORE risk tables were applied [6]. Additional studies have also confirmed the suboptimal performance of the SCORE algorithm to identify patients with RA at high risk of CVD disease [7].

Given these findings, it is clear that new and updated guidelines are needed. Interestingly, in 2021, a new algorithm, SCORE2 was derived, calibrated, and validated, then launched in an effort to predict the 10-year risk of first-onset CV disease in European populations [8]. This new calculator improves the identification of people at high risk of developing CV disease because SCORE2 has been recalibrated using the most contemporary and representative CVD rates available. Moreover, SCORE2 now accounts for the impact of competing risks by non-CVD deaths, whereas SCORE did not.

The performance of SCORE2 in patients with RA is unknown. In the current work, we compared the predictive capacities of the original SCORE and the new SCORE2 to identify RA patients with subclinical atherosclerosis who are, consequently, at high risk of CVD.

## 2. Materials and Methods

### 2.1. Study Participants

Our study had a cross-sectional design and recruited 1168 subjects with RA. Only patients 40 years or older were included in the current study. All of them fulfilled the 2010 ACR/EULAR classification criteria for RA [9]. Our study was performed between 2012 and 2019; disease duration needed to be ≥ 1 year. Patients under prednisone treatment were permitted to participate in the study only if they were taking ≤ 10 mg/day. Exclusion criteria were: diabetes, a glomerular filtration rate < 60 mL/min/1.73 m^2^, the presence of prior CV events, a history of cancer, active infection or the existence of other inflammatory chronic disease. The study protocol was approved by the Institutional Review Committees at Hospital Marqués de Valdecilla, Spain (Approval reference: 17/2012). Research was carried out in compliance with the Declaration of Helsinki. All subjects provided informed written consent.

### 2.2. Data Collection

RA patients included in the present work completed a questionnaire on medication use and CV risk factors and underwent a physical examination. Body-mass index (the weight in kilograms divided by the square of the height in meters), abdomen circumference and systolic and diastolic blood pressure were assessed under standardized conditions. Smoking status (current smoker versus non-smoker) was recorded. Medical registers were reviewed shortly after the visit, to verify specific medications or diagnoses. Disease activity in patients with RA was measured using the following scores, as previously described: Disease Activity Score (DAS28) in 28 joints [10], the Clinical Disease Activity Index (CDAI) [11] and the Simple Disease Activity Index (SDAI) [12].

The SCORE and SCORE2 were calculated as described elsewhere [5,8]. SCORE has been classically categorized in low (<1%), moderate (1–4%), high (5–9%) or very high (>10%) risk categories. In contrast, the 2021 European Society of Cardiology Guidelines on cardiovascular disease prevention in clinical practice [13] proposed that the SCORE2 risk categories be reduced to three (low to moderate, high and very high) and that each category use different numerical cutoff levels depending on different age groups (<50, 50–69 and ≥70 years). In addition, SCORE calculated the 10-year risk of death from CV disease. However, since CV disease morbidity, when combined with CV disease mortality, better reflects the total burden of atherosclerotic CV disease, SCORE2 now estimates an individual’s 10-year risk of fatal and non-fatal CV disease events in individuals aged 40 to 69 years. For healthy people aged ≥70 years, the SCORE2-OP (older persons) algorithm estimates 5-year and 10-year fatal and non-fatal CV disease events.

### 2.3. Carotid Ultrasound Assessment

A carotid ultrasound examination was used to assess carotid intima-media thickness (cIMT) at least 5 mm below the end of the common carotid artery, and to detect focal plaques in the extracranial carotid tree [14]. Ultrasound was performed by a single rheumatologist with experience in the technique. The Esaote Mylab 70 (Esaote SPA, Genoa, Italy) was used. Additionally, cIMT was measured using a software-guided radiofrequency technique (Quality Intima Media Thickness (QIMT, Esaote, Maastricht, Holland)). For the definition of carotid plaque, we based our work on the Mannheim consensus plaque criteria: a protrusion at least 50% greater than the surrounding cIMT, a focal protrusion in the lumen measuring at least cIMT > 1.5 mm or arterial lumen encroaching > 0.5 mm [15].

### 2.4. Statistical Analysis

For binary variables, clinical characteristics were shown as frequencies. Continuous variables data were disclosed as mean ± standard deviation (SD); in the case of non-normally distributed variables, median and interquartile range (IQR) were shown. Correlations between continuous variables were studied using Spearman’s Rho correlation coefficients. Fisher r-to-z transformation was used for the comparison between correlation indexes. The relationships of SCORE and SCORE2 to the presence of carotid plaque in patients with RA were analyzed using receiver-operating characteristic curves (ROC) (through the relationship of sensitivity versus 1-specificity). Areas under the curves (AUC), derived from the same cases, were compared using the method of DeLong et al. [16]. Regarding missing data handling, missing data were omitted and the remaining data were analyzed (listwise deletion). No imputation of missing data was performed in our statistical analysis. Analyses were performed using SPSS software, version 26 (IBM, Chicago, IL, USA). A *p*-value < 0.05 was considered statistically significant.

## 3. Results

### 3.1. Demographic, Laboratory and Disease-Related Data

A total of 1168 patients with RA were included in this study. Demographic and disease-related characteristics of the participants are shown in Table 1. Most of the patients were women (76%) and the mean age ± SD was 60 ± 11 years. Twenty-five of the patients were current smokers, 39% had hypertension, and 51% fulfilled the definition for dyslipidemia. Although patients who had had CV events were excluded, some patients were taking preventive statins (26%) (Table 1). Regarding disease-related data, disease duration was 6 (IQR 2–12) years and disease activity scores showed that most of the patients had a low-moderate disease activity at the time of recruitment. Additionally, half of the patients (50%) were taking prednisone (the median dose of those on prednisone was 5 [IQR 5–7.5] mg/day at the time of the study). In total, 60% percent and 55% of the patients were found to be positive for rheumatoid factor and ACPA, respectively. DMARD (disease-modifying antirheumatic drug) use was reported in 76% of the patients, and 58% were taking methotrexate at the time of the study. Regarding subclinical carotid atherosclerosis, the mean cIMT was 707 ± 141 microns, and 58% of the patients had carotid plaque. For additional information on the characteristics of RA patients, see Table 1.

### 3.2. SCORE2 and SCORE Relationship with Each Other and with Carotid Plaque and cIMT

Absolute values of SCORE and SCORE2 were 1.6 (IQR 0.6–4.0) and 4.2 (IQR 2.3–7.1), respectively. Both calculators correlated strongly between them (Spearman’s Rho = 0. 961, *p* < 0.001).

**Figure 1:**
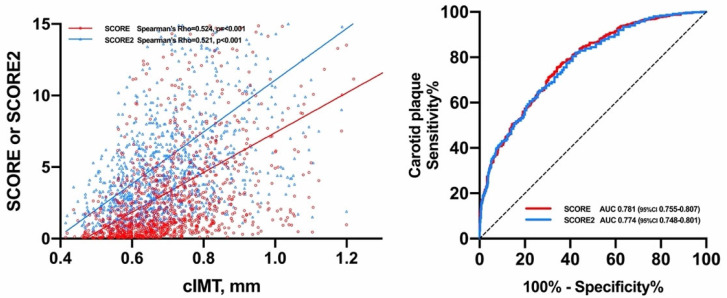
Relationship of SCORE and SCORE2 with cIMT and carotid plaque.

Both SCORE (Spearmen’s Rho = 0.524) and SCORE2 (Spearman’s Rho = 0.521) were similarly correlated with cIMT. In this regard, no significant difference was found between the two correlation values (*p* = 0.92). Likewise, both calculators showed significant discrimination for the presence of carotid plaque: SCORE AUC 0.781 (95%CI 0.755–0.807) and SCORE2 AUC 0.774 (95%CI 0.748–0.801). The difference between AUCs did not yield statistical significance (*p* = 0.14) (Figure 1).

**Figure 2:**
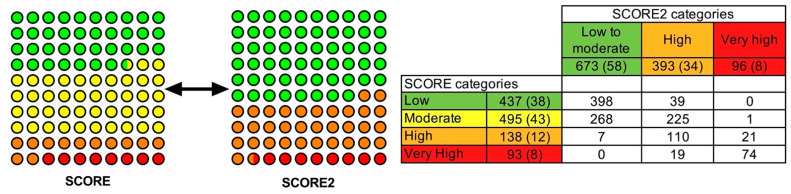
Differences in the distribution of CV risk categories between SCORE and SCORE2 calculators.

According to SCORE, 437 (38%) patients with RA were distributed in low CV risk, 495 (43%) in moderate CV risk, 138 (12%) in high CV risk and 93 (8%) in very high CV risk category, respectively. Therefore, when the original SCORE was applied, 80% of the patients with RA were included in the low or moderate CV risk categories and 20% in the high or very high CV risk categories. On the contrary, using SCORE2, the stratification of the patients differed; 673 (58%) of the patients were included in the low or moderate CV risk category, 393 (34%) in the high risk category and 96 (8%) in the very high CV risk category. Consequently, according to the SCORE2, 58% of the patients were in the low or moderate CV risk category and 42% in the high and very high CV risk category (Figure 2). The number of patients included in the high and very CV risk category showed significant differences between SCORE and SCORE2 (*p* < 0.001).

In summary, if low and moderate and high and very high categories CV risk categories were considered as the only two groups in both calculators, 932 (80%) and 231 (20%) subjects, respectively, would have been in the low or moderate and high or very high categories using SCORE, and 673 (58%) and 489 (42%), respectively, using SCORE2. Therefore, the percentage of patients in each category in both calculators was found to be statistically significantly different (*p* < 0.001).

## 4. Discussion

In our study, the use of SCORE2 did not show a greater association with the presence of subclinical atherosclerosis in patients with RA than the original SCORE. In this sense, the association of SCORE and SCORE2 with cIMT and the presence of carotid plaque was not different between the two calculators. However, the new categorization into CV risk groups proposed in the SCORE2 does represent a great advance in the identification of patients with RA at high risk of CV disease. Using SCORE2, the number of patients with high or very high CV risk went from 20% to 42%. This was deemed clinically relevant as it implied that, with this new version, a greater number of patients with RA would benefit from stricter CV risk control strategies.

Identifying patients with RA at high CV risk and preventing CV events is an important goal for rheumatologists. Current CV risk calculation tools have previously been described as having suboptimal performance in patients with RA [7,17,18]. For example, a prior work that aimed to assess the predictive capacity of four generic CV risk models for fatal and non-fatal CV events in patients with RA established that some risk models underestimated (SCORE, Framingham score, Reynolds score) while others overestimate (QRisk II) CV risk in RA patients [7]. This is believed to be a consequence of the fact that each of these CV risk prediction tools is largely based on values related to traditional CV risk factors; they do not take into account other disease-related variables, such as disease activity, inflammation, the use of therapies such as glucocorticoids or the genetic component associated with the disease. Furthermore, systematically performing carotid ultrasound or other surrogates of CV risk like coronary artery calcium has been proven to allow reclassification of CV risk in patients with RA because the presence of, for example, carotid plaque causes patients to be reclassified to very high CV risk [14]. Certainly, we believe that since SCORE and SCORE2 showed a similar correlation with subclinical atherosclerotic disease in our cohort of RA patients, SCORE2 would likely remain a poor tool in RA patients. However, studies with a prospective design are needed to confirm this hypothesis.

We recognize the limitation that SCORE was developed for the prediction of CV events and not for subclinical arteriosclerosis. However, it must be taken into account that subclinical carotid arteriosclerosis has been shown to be strongly related to future CV events, not only in the general population [19] but also in other inflammatory diseases such as RA [4]. We also recognize as a potential limitation that a subgroup existed comprised of RA patients that were under CVD preventive medications such as statins, aspirin and antihypertensives. We believe that the usage of such treatments could have altered the absolute value of the CV calculators. Nevertheless, it has to be taken into account that our proposal was to assess the performance of the two scores in a real-world setting and not merely in patients naïve to CVD prevention medications. Additionally, carotid ultrasounds for this work were performed by a single rheumatologist. This has not allowed us to calculate interobserver concordance tests regarding the presence of carotid plaque or cIMT measurement. We also believe that the high number of patients recruited in our study (more than a thousand) strengthened our findings.

The fact that SCORE2 was developed in datasets of contemporary populations and that its accuracy and validity are superior have led us to propose its generalizability in patients with RA.

In conclusion, the predictive capacity of SCORE2 for the presence of subclinical atherosclerosis is similar to that of SCORE. However, the use of SCORE2 makes it possible to identify a significantly higher proportion of RA patients with high or very high CV risk. Taking this finding into account, we recommend the use of this new SCORE2 calculator for CV risk stratification of patients with RA.

## Figures and Tables

**Table 1:** Demographics, cardiovascular risk factors, and disease-related data in RA patients.

		RA	
		(n = 1168)	Missing (n, %)
Age, years		60 ± 11	3 (0)
Women, n (%)		887 (76)	1 (0)
BMI, kg/m^2^		28 ± 5	7 (1)
Abdominal circumference, cm		94 ± 16	0 (0)
Cardiovascular data			
CV risk factors, n (%)			
Current smoker		293 (25)	1 (0)
Obesity		335 (29)	4 (0)
Dyslipidemia		592 (51)	1 (0)
Hypertension		460 (39)	1 (0)
Diabetes Mellitus		0 (0)	-
Blood pressure, mm Hg			
Systolic		132 ± 17	19 (2)
Diastolic		79 ± 10	20 (2)
Lipids			
Total cholesterol, mg/dL		205 ± 36	4 (0)
Triglycerides, mg/dL		114 ± 62	4 (0)
HDL-cholesterol, mg/dL		61 ± 17	5 (0)
LDL-cholesterol, mg/dL		121 ± 31	8 (1)
Atherogenic index		3.6 ± 1	5 (0)
Statins, n (%)		300 (26)	1 (0)
Disease related data			
Disease duration, years		6 (2–12)	0 (0)
CRP at time of study, mg/L		2.8 (1.0–6.9)	0 (0)
ESR at time of study, mm/1st hour		13 (6–24)	254 (22)
Rheumatoid factor, n (%)		693 (60)	5 (0)
ACPA, n (%)		621 (55)	45 (4)
History of extraarticular manifestations, n (%)		220 (20)	62 (5)
Erosions, n (%)		621 (60)	130 (11)
DAS28-ESR		3.14 ± 1.49	17 (1)
DAS28-PCR		3.03 ± 1.28	19 (2)
SDAI		10 (5–19)	0 (0)
CDAI		9 (4–16)	12 (1)
HAQ		0.750 (0.250–1.250)	470 (40)
Current drugs, n (%)			
Prednisone		585 (50)	1 (0)
Prednisone doses, mg/day		5 (5–7.5)	0 (0)
NSAIDs		457 (39)	1 (0)
DMARDs		889 (76)	1 (0)
Methotrexate		671 (58)	1 (0)
Leflunomide		135 (12)	1 (0)
Hydroxychloroquine		258 (22)	1 (0)
Salazopyrin		42 (4)	1 (0)
Anti TNF therapy		151 (13)	1 (0)
Tocilizumab		56 (5)	2 (0)
Rituximab		19 (2)	2 (0)
Abatacept		22 (2)	1 (0)
Baricitinib		10 (1)	1 (0)
Tofacitinib		14 (1)	1 (0)
Subclinical atherosclerosis			
Carotid IMT, microns		707 ± 141	6 (1)
Carotid plaques, n (%)		674 (58)	4 (0)

Data represent mean ± SD or median (IQR) when data were not normally distributed. CV: cardiovascular; LDL: low-density lipoprotein; HDL: high-density lipoprotein; CRP: C reactive protein. cIMT: carotid intima media thickness; HAQ: Health Assessment Questionnaire. NSAID: Nonsteroidal anti-inflammatory drugs; DMARD: disease-modifying antirheumatic drug. TNF: tumor necrosis factor; ESR: erythrocyte sedimentation rate. BMI: body mass index; DAS28: Disease Activity Score in 28 joints. DAS28: Disease Activity Score in 28 joints; ACPA: Anti-citrullinated protein antibodies. CDAI: Clinical Disease Activity Index; SDAI: Simple Disease Activity Index. RA: Rheumatoid arthritis. No diabetic patients were included, so missing data does not apply for diabetes.

## Data Availability

The data underlying this article will be shared upon reasonable request to the corresponding author.
