# Correction: Ferraz-Amaro et al. SCORE2 Assessment in The Calculation of Cardiovascular Risk in Patients with Rheumatoid Arthritis. *Diagnostics* 2021, *11*, 2363

**DOI:** 10.3390/diagnostics12020521

**Published:** 2022-02-18

**Authors:** Iván Ferraz-Amaro, Alfonso Corrales, Belén Atienza-Mateo, Nuria Vegas-Revenga, Diana Prieto-Peña, Julio Sánchez-Martín, Cristina Almeida, Juan Carlos Quevedo-Abeledo, Ricardo Blanco, Miguel Á. González-Gay

**Affiliations:** 1 Division of Rheumatology, Hospital Universitario de Canarias, 38320 Tenerife, Spain; 2 Internal Medicine Department, University of La Laguna, 38071 Tenerife, Spain; 3 Division of Rheumatology, Hospital Universitario Marqués de Valdecilla, Universidad de Cantabria, 39008 Santander, Spain; afcorralesm@hotmail.com (A.C.); mateoatienzabelen@gmail.com (B.A.-M.); nuriavegas2@gmail.com (N.V.-R.); diana.prieto.pena@gmail.com (D.P.-P.); jsm132@hotmail.com (J.S.-M.); rblancovela@gmail.com (R.B.); 4 Epidemiology, Genetics and Atherosclerosis Research Group on Systemic Inflammatory Diseases, Hospital Universitario Marqués de Valdecilla, IDIVAL, 39011 Santander, Spain; 5 Division of Rheumatology, Hospital Doctor Negrín, 35010 Las Palmas de Gran Canaria, Spain; almeidasantiago.cristina@gmail.com (C.A.); quevedojcarlos@yahoo.es (J.C.Q.-A.); 6 Cardiovascular Pathophysiology and Genomics Research Unit, School of Physiology, Faculty of Health Sciences, University of the Witwatersrand, Johannesburg 2000, South Africa

In the original article [1], there was a mistake in Figure 1 as published. Due to an error in the proofreading stage, ROC curves in that figure do not represent the exact curves. The corrected Figure 1 appears below. The authors apologize for any inconvenience caused and state that the scientific conclusions are unaffected. The original publication has also been updated.

## Figures and Tables

**Figure 1:**
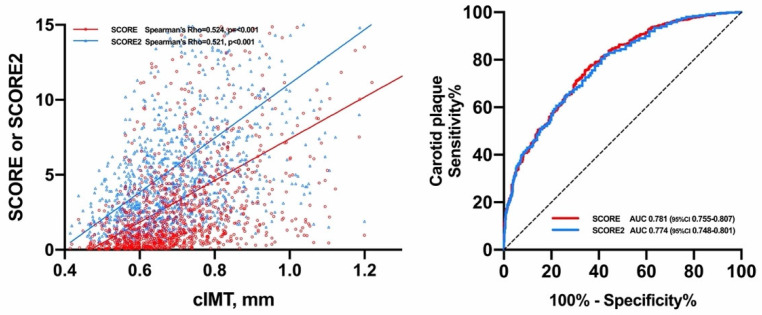
Relationship of SCORE and SCORE2 with cIMT and carotid plaque.
